# Divergent Rules for Pollen and Nectar Foraging Bumblebees – A Laboratory Study with Artificial Flowers Offering Diluted Nectar Substitute and Pollen Surrogate

**DOI:** 10.1371/journal.pone.0091900

**Published:** 2014-03-17

**Authors:** Sabine Konzmann, Klaus Lunau

**Affiliations:** Institute of Sensory Ecology, Heinrich-Heine-University Düsseldorf, Düsseldorf, Germany; Royal Holloway University of London, United Kingdom

## Abstract

Almost all bees collect nectar and pollen from flowers. Female bees collect pollen to provision their nest cells, whereas they use nectar for individual energy supply and nest cell provisioning. Bees fine-tune nectar foraging to the amount and to the concentration of nectar, but the individual bees' response to variability of amount and concentration of pollen reward has not yet been studied thoroughly in laboratory settings. We developed an experimental set-up in which bumblebees simultaneously collected sugar solution and pollen from artificial flowers; natural pollen was mixed with cellulose powder or glass powder as a pollen surrogate. Here we show that bumblebee (*Bombus terrestris*) workers do not specialise in nectar or pollen collection, but regularly collect both rewards on the same day. When offered a fixed pollen reward and varied amounts and concentrations of sugar solution, the bumblebees fine-tuned sugar solution foraging dependent on both the volume and concentration, with strong preferences for the highest concentration and the greatest volume. In the reciprocal tests, when offered a fixed sugar reward and varied amounts and concentrations of pollen mixed with a nutrient-free pollen surrogate, the bumblebees follow more an all-or-none rule for pollen, accepting all amounts and concentrations except pure surrogate. It is discussed how the bumblebees' ability to sense sugar, and their apparent inability to sense the pollen protein content, shaped their foraging behaviour. It is argued that the rarity of nectar mimicry and the frequency of pollen mimicry in natural flowers might be interpreted in the context of divergent abilities of nectar and pollen recognition in bees.

## Introduction

For social bees, the gathering of nectar and pollen is essential for the maintenance and growth of the colony [1]. Nectar is the source of energy for the queen and the workers, whereas pollen supplies protein for the developing larvae, freshly emerged workers and the queen [2]–[4]. Nectar foraging has been thoroughly studied, particularly in honeybees [5], [6]; however, these studies mostly focus on recruitment behaviour [7], [8], division of labour [9]–[14], learning [15]–[17] and mathematical modelling [18]–[21] but rarely studying nectar foraging in comparison to pollen foraging. Tests have shown that foraging for nectar and pollen in eusocial bees is regulated by storage levels [14], [22]–[25] to different degrees. When nectar reward is declining, bumblebees are more ready than honeybees to search for new nectar sources [26]. However when the colony has some nectar and pollen stores, it is not known how the worker bees allocate their nectar- and pollen-foraging activities related to the quantity and quality of the available floral resources.

In this study, we examined for the first time the pollen and nectar collecting behaviour of individual bumblebees (*Bombus terrestris*) with respect to different quantities and qualities of both main floral resources when offered simultaneously in an array of artificial flowers. While it is known that the bumblebee colony – like all social insect colonies – is based on the division of labour [27], it is not clear whether workers specialize in foraging for only one resource and how pollen and nectar foraging are interdependent. However it has been shown that the division of labour in honeybees is characterized by changes in the tasks they perform as they age [28] and that bumblebees are more able to forage on protein-rich pollen sources than honeybees [29]. Besides collecting nectar, all worker bees are morphologically and behaviourally able to collect pollen and carry pollen loads back to the nest. The flowers of many plants offer both nectar and pollen. Nectar is collected exclusively by the nectar-sucking activity of the workers, whereas pollen is collected actively by buzzing [30] or other movements [31] as well as passively during flower handling. Bees can assess the nectar reward by simply sensing the sugar molecules [32], [33], whereas the bees' capabilities to assess pollen sources while foraging are not well understood. Although pollen specialist bees can identify the pollen of their host plant species or family by odour [34], no general key substance of pollen has been identified [35]. The free amino acid proline is very common in pollenkitt [31], [36], [37] and represents a potential chemical cue used by syrphid flies to identify pollen [38]. Despite the fact that bees recognise and prefer nectar enriched with proline [39] there is no report about proline being a key substance of pollen detection for bees. Although bees might be able to taste proline they are unable to smell proline at concentrations that occur in flowers [40]. The apparent inability of bees to assess pollen reward is probably due, at least in part, to the fact that the protein molecules are deposited in pollen grains which are completely covered by a resistive wall of sporopollenin [31], [41]. This means that nutrients can only be sensed by bees directly if pollen grains are broken open by the mandibles or during digestion [42]. Based on this knowledge, we infer that bumblebee workers can more easily discriminate between different qualities of nectar than of pollen.

We set up laboratory experiments to compare bumblebee foraging rules for nectar and pollen and their ability to respond to variation in both the quantity and quality of rewards. First, we monitored the foraging specialization of individual bumblebees with respect to pollen and nectar substitute over several weeks. Second, we assessed the forager responses to being simultaneously offered variation in nectar and pollen quality. Nectar reward was simulated using a sugar solution diluted with water, and pollen reward was simulated by washed and dried honeybee-collected pollen diluted with dyed cellulose powder. Third, we observed the pollen and nectar foraging behaviour of individual bumblebees in an array of artificial flowers each offering either nectar or pollen. Nectar reward was simulated using a sugar solution diluted with water and the pollen reward was hand-collected *Pinus* pollen diluted with glass powder. We tested 2×2 reciprocal foraging scenarios: 1) fixed nectar reward and pollen reward varying in pollen quantity, 2) fixed nectar reward and pollen reward varying in pollen quality, 3) fixed pollen reward and nectar reward varying in nectar quantity and 4) fixed pollen reward and nectar reward varying in nectar quality. As both, cellulose powder and glass powder are odourless, inert and indigestible substances we were able to use this experimental paradigm to determine the bumblebees' response to amount and concentration of sugar and pollen. We were particularly interested in testing the hypothesis that the bumblebees' apparent inability to sense the nutitional value, i.e. the protein content of pollen grains, affects their pollen-foraging behaviour as compared to their nectar-foraging behaviour. This hypothesis predicts that bumblebees respond more sensitively to varying sugar concentrations than pollen concentrations.

## Materials and Methods

### Bumblebee keeping

**Figure 1:**
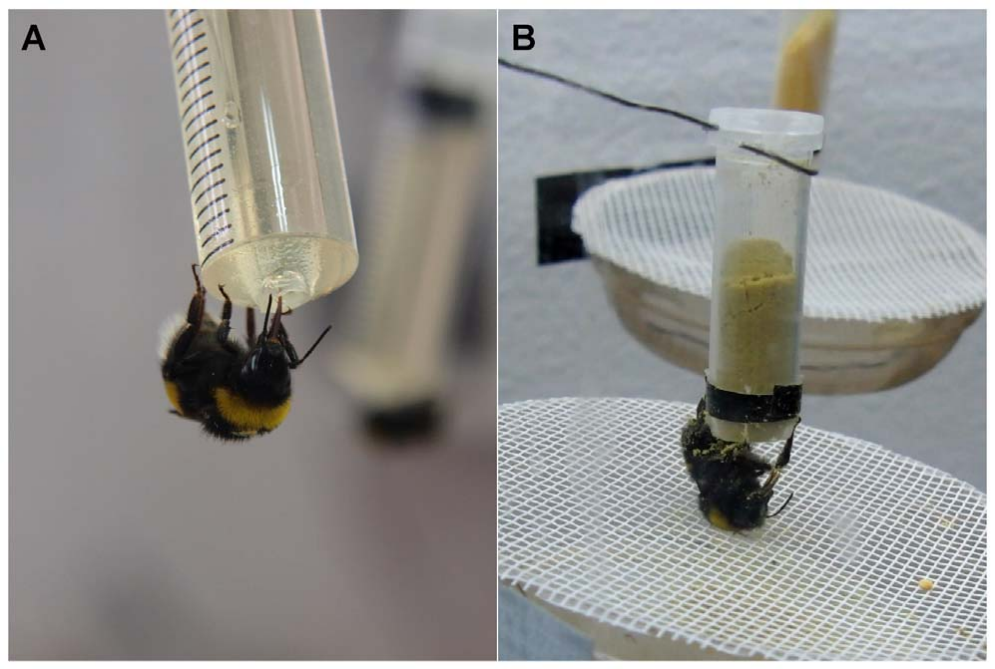
Bumblebees collecting resources from artificial feeders. **A**: Bumblebee drinking sugar solution from a 5 ml syringe with a cut tip. More than one worker can collect sugar solution from one feeder simultaneously. **B**: Bumblebee gathering pollen from a modified Eppendorf tube. By buzzing while placed underneath the tube, the worker loosens pollen from the tube which accumulates on her ventral side from whence she is able to pack it into her corbiculae.

The study was conducted from January to September 2011 with three successive laboratory colonies of the buff-tailed bumblebee (*Bombus terrestris*). These were obtained from commercial breeders; the first one from Koppert B.V. (Netherlands) and the subsequent two from re-natur GmbH (Germany). The colonies were delivered in a plastic cage set in a cardboard box ready for placement. This box was connected to a transparent Perspex feeding box (L×W×H = 40×40×80 cm) by a transparent Perspex tunnel. The bumblebees were fed 30% sugar solution (diluted Apiinvert and Biogluc respectively, included in the colony delivery and consisting of a sugar solution, a preservative and a colouring agent) by transparent 5 ml plastic syringes accessible to foragers only by flying (Fig. 1). Honeybee-collected pollen was washed three times with water, air-dried and offered as a dry powder in Eppendorf tubes. To render the pollen accessible to the bees, the bottom of the tube was cut and the aperture was barred with pieces of thread which were fixed to the tube with tape. A barred dish was placed underneath the feeder to collect surplus pollen and to enable the bees to land (Fig. 1). Bumblebee workers collecting pollen or sugar solution were captured and individually marked with numbered Opalith tags.

### Experimental setups

#### Individual foraging tasks

In this experiment the nectar- and pollen-foraging activities in the feeding box of 48 individually marked foragers of the first colony were monitored for 41 days between 4^th^ February and 27^th^ March in 2011. The daily observation time was between 11:00 and 18:00 CEST continuously. In addition, the foraging flights of the 48 bumblebee workers were counted for 1 hour (from 11:00–12:00) on 5 different days. Food, sugar solution (27% by weight) as well as washed and dried honeybee-collected pollen, was provided to bees at 11:00. The amount of sugar solution and pollen offered was adjusted to colony storage levels to prevent the bumblebees from overstocking either resource, varying between 10 and 25 ml of sugar solution and 1 and 2 g of pollen, respectively. Supply of pollen and sugar solution was reduced, if more than approximately 10% of the visible nest cells were filled with pollen and honey respectively. Although nectar forager recruitment decreases when more than 5% of the visible honeypots are filled [43], a higher threshold was chosen as observation had shown that pollen was only very rarely collected while less than 5% of the visible honeypots were filled. While honey stores were higher than approximately 5%, more bumblebees collected pollen which was a prerequisite for our tests. Since nest cells were quickly filled with honey, the 5 nectar feeders were offered consecutively with only 2 feeders at one time in order to provide sugar solution during most of the observation time. The 5 pollen feeders were offered simultaneously and refilled after being emptied by the bumblebees, because only relatively few nest cells were filled with pollen. Although the colony was beginning to decline, the queen was alive and laying (few) eggs until the end of the observation period. No young queens (gynes) or males were produced.

#### Collective foraging on resources varying in quality

**Figure 2:**
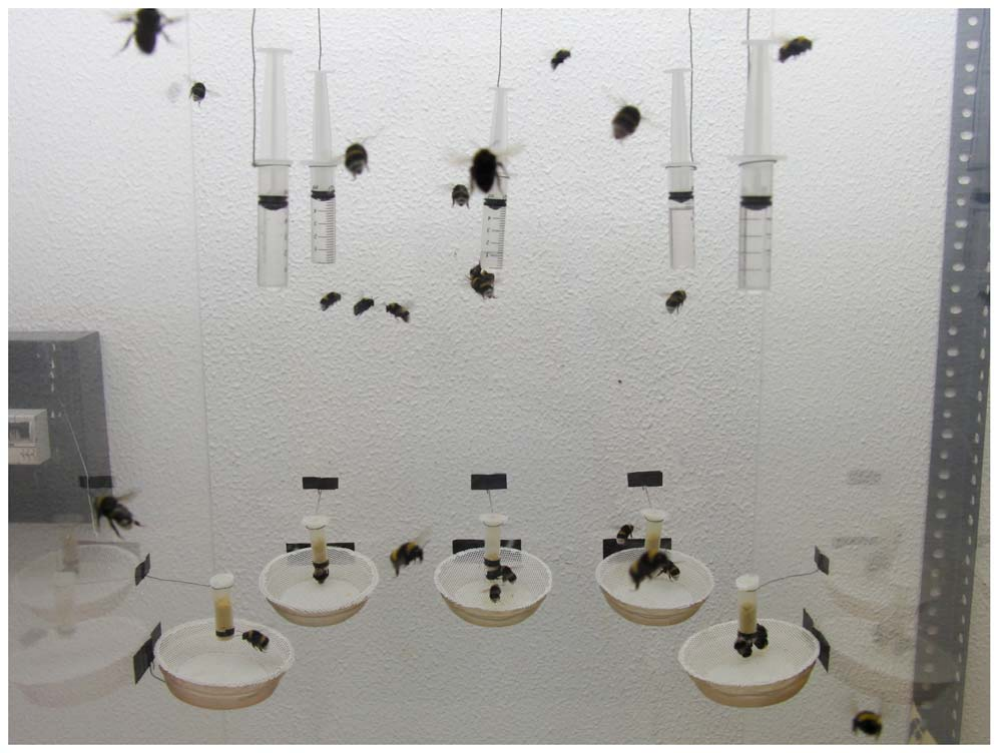
Experimental set-up for bees able to collect both sugar solution and pollen from artificial feeders. Bumblebees foraging for sugar solution from modified syringes and pollen from feeders in the feeding box. Diluted sugar solution is offered in following concentrations (from left to right: 0%, 18%, 36%, 27%, 9%). Blends of washed honeybee-collected pollen and cellulose powder are offered in following concentrations (from left to right: 0%, 25%, 100%, 75%, 50%).

In this experiment the second bumblebee colony was offered 5 nectar feeders and 5 pollen feeders simultaneously in the feeding box (Fig. 2) from 11:00 for a varying length of time in the course of one month. The nectar feeders were modified syringes and offered 5 ml of sugar solution each in various concentrations. Syringes with 36%, 27%, 18%, 9% and 0% sugar solution (per cent by weight; Biogluc diluted with water) was offered simultaneously. The pollen feeders (described above) were plastic dispensers combined with a dish covered by a gauze in order to accumulate any pollen lost during pollen collection by the worker bees, and offered 1 ml of pollen each in different concentrations (100%, 75%, 50%, 25% and 0% pollen percent by volume; washed and dried honeybee-collected pollen diluted with cellulose powder previously dyed with sugar-free food colouring to resemble the colour of pollen) was offered simultaneously. Washed honeybee-collected pollen was used as it contains a variety of pollen suitable to provide the colony with protein. Cellulose powder was used because larval development would not be impaired even if workers collected it in large amounts and it has been used in previous studies to produce varying pollen qualities [25], [44]. The weight per unit volume of washed and dried pollen exceeded that of cellulose powder by a factor of 4 (the specific weight of washed bee-collected pollen was 0.5 g/ml; that of dyed cellulose powder amounted to 0.125 g/ml). In two tests, the amount of sugar solution and pollen surrogate, respectively, collected from each feeder was measured at different points in time: To test whether bumblebees are able to distinguish, and hence prefer, the highest resource quality we interrupted foraging and thus ended the trial when the bumblebees had just emptied the first feeder and measured the amount of both resources collected. Following this, all feeders were replenished to start the next trial: 76 trials on 14 non-successive days for sugar solution and 12 trials on five non-successive days for pollen. To ascertain whether bumblebees differentiate between different concentrations of resources, we measured the amount of each resource left in the feeders when the bumblebees ceased collecting it after several hours of foraging *ad libitum*. A total of 11 trials for sugar solution and 3 trials for pollen were performed towards the end of the daily foraging period (after several trials in which foraging was interrupted when the first feeder was emptied); again, all of the feeders were refilled before the trial.

#### Individual foraging on resources varying in both quality and quantity

**Figure 3:**
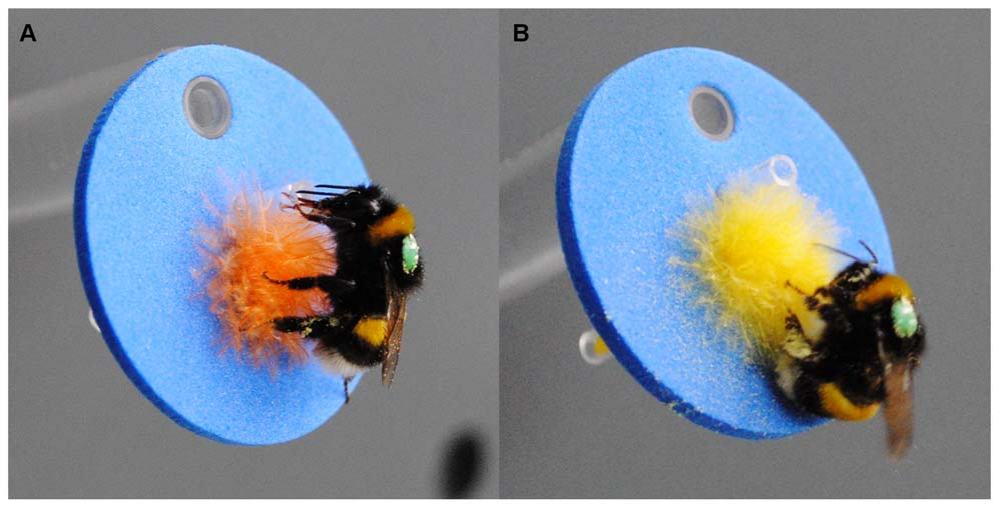
Bumblebees gathering resources from artificial flowers. **A**: Bumblebee collecting sugar solution from an artificial flower. The worker gathers sugar solution from a transparent plastic tube right above the orange-coloured feathers. **B**: Bumblebee buzz-collecting pollen from a bunch of yellow feathers; note the blurring of the wings caused by vibrations.

This experiment was performed over three months with individual bumblebee workers from the second and third colony using a vertical artificial “flower meadow”, a grey polyvinyl chloride (PVC) wall on which 42 artificial flowers were presented. Individual workers on their way to the feeding box were taken from the tunnel and allowed to forage on the flower meadow containing 21 yellow and 21 orange flowers (Fig. 3) *ad libitum* (which took up to 18 minutes) before being released into the tunnel to return to the nest. We only tested workers that had previously been observed foraging for sugar solution or pollen in the feeding box on the same day. The artificial flowers (henceforth called flowers) were made of circular blue EVA foam sheet (Ø 30 mm) equipped with a small bunch of yellow or orange coloured chick feathers and a cut pipette tip. Flowers with a bunch of yellow feathers offered pollen in variable quantity or quality, applied onto the feathers, whereas flowers with orange coloured feathers offered nectar in variable quantity or quality contained in the cut pipette tip. The spatial arrangement of the two flower types on the wall was pseudo-randomized. Previously visited flowers were not refilled during the trial but replaced by fresh ones after each trial. Four series of tests were carried out, each of them comprising five tests with different quantities or qualities of one resource and a standard reward of the other resource (Table 1). In the two series of tests in which nectar quantity and nectar quality were varied, 1 ml of pure *Pinus* pollen (i.e. ∼0.05 ml pollen per flower) was used as a standard “pollen” reward. The nectar quantities tested were 210 μl; 105 μl; 52.5 μl; 21 μl; 0 μl of 45% sugar solution (i.e. 10 μl; 5 μl; 2.5 μl; 1 μl; 0 μl per flower). The nectar qualities tested were 60%; 45%; 30%; 15%; 0% sugar solution diluted with water; the original Biogluc sugar solution was 60%. In the two series of tests in which pollen quantity and pollen quality were varied, 210 μl of 45% sugar solution (i.e. 10 μl sugar solution per flower) was used as a standard nectar reward. The pollen quantities tested were 1 ml, 0.5 ml, 0.25 ml, 0.1 ml, 0 ml of pure *Pinus* pollen evenly distributed to all of the 21 flowers. The specific weight of *Pinus* pollen was 0.4 g/ml. The pollen qualities tested were 100%, 75%, 50%, 25%, 0% of *Pinus* pollen diluted with glass powder (Worf Glaskugeln GmbH). The glass beads had an average diameter of 50 μm. A blend of *Pinus* pollen and glass beads has been used in previous studies (Lunau & Piorek, Lunau & Goertz, both unpublished) and was used in this experiment as it could easily be applied to the feathers of the artificial flowers and the bumblebees could pack it into their corbiculae more easily than the blend of washed bee-collected pollen and cellulose powder. Although *Pinus* is primarily wind-pollinated, bumblebees collected its pollen from a feeder or an artificial flower. It is also known that honeybees readily collect *Pinus* pollen [45]. The development of the bumblebee colony was obviously not affected by collecting *Pinus* pollen by some workers, probably because the colony was also fed with honeybee collected pollen and/or the nutritional value of *Pinus* pollen is sufficient.

**Table 1:** Combinations of rewards in the individual foraging on resources varying in both quality and quantity experiment.

Series of tests	Varied reward	Standard reward
Sugar solution quantity	210 μl; 105 μl; 52.5 μl; 21 μl; 0 μl sugar solution (45%)	1 ml 100% pollen
Sugar solution quality	60%; 45%; 30%; 15%; 0% sugar solution (210 μl)	1 ml 100% pollen
Pollen quantity	1 ml; 0.5 ml; 0.25 ml; 0.1 ml; 0 ml pollen (100%)	210 μl 45% sugar solution
Pollen quality	100%; 75%; 50%; 25%; 0% pollen (1 ml)	210 μl 45% sugar solution

Quantity and quality of sugar solution and pollen rewards offered simultaneously in four series of tests, each of them comprising five tests with a varied reward – different quantities or qualities – of one resource and a standard reward of the other resource.

In total 41 workers were tested in 199 individual trials (mean trials/ worker  = 4.85±0.72 SE, maximum number of trials/ worker  = 16 different trials) meaning that each worker was not tested for all combinations of rewards. In their first trial each bumblebee was initially released at an orange flower offering sugar reward, learning in the course of their first trial – by visiting both colours of flowers – that orange coloured flowers offered sugar solution and that yellow flowers offered pollen. All bees visited both colours of flowers at least once in their first trial. For each trial, we recorded the number of flowers of each type visited, i.e. every time during a bumblebee tasted, sampled or collected the reward offered by the flower. Approach flights, without direct contact to the reward, were not counted. After each trial, we calculated the amount of rewards collected by measuring the sugar solution left in the pipette tips and weighing the pollen taken from one of the bee's corbiculae (this assumes that the pollen quantity is the same in both corbiculae).

We analysed the data of the collective foraging on resources varying in quality experiment using Anova and Tukey's Hsd-test in R (v2.12.1) [46]. The individual foraging on resources varying in quality and quantity experiment was analysed using repeated measures Anova and pairwise comparisons using paired t-tests with Bonferroni correction.

## Results

### 

#### Individual foraging tasks

**Figure 4:**
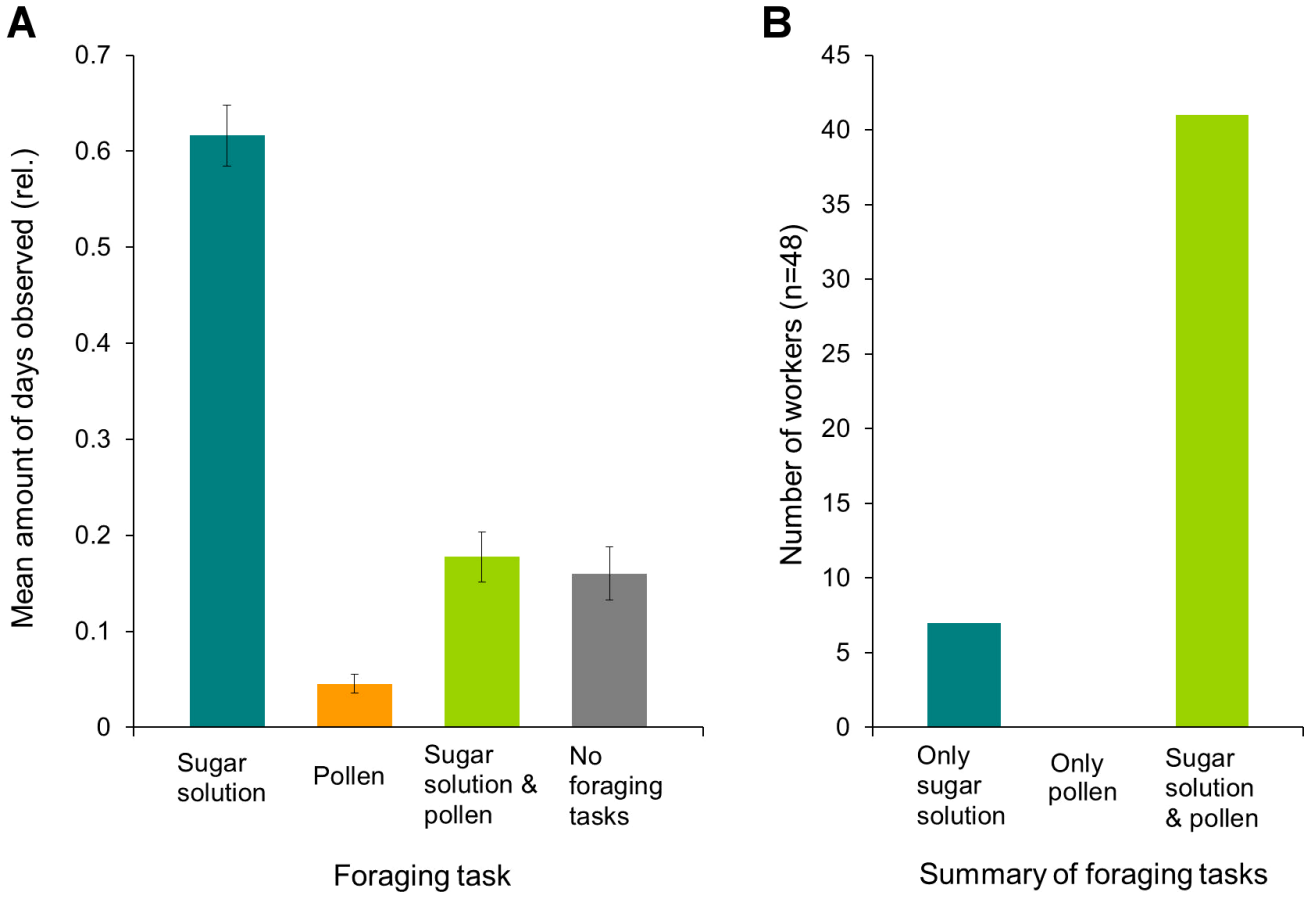
Summary of foraging behaviour for 48 tagged bumblebee workers. **A**: Mean amount of days that the bumblebee workers perform specific foraging tasks. Each bumblebee was observed on 20–41 days (mean number of days observed  = 26.52±1.29 SE). Note that while collecting pollen, bumblebees also gathered small amounts of sugar solution to pack the pollen; the column “pollen” thus excludes foraging bouts just for sugar solution. **B**: Summary of all foraging tasks performed by each bumblebee in the course of the observation period. 7 bumblebees only ever collected sugar solution, 41 bumblebees collected sugar solution, pollen, or both sugar solution and pollen on different days.

We monitored the foraging behaviour of 48 individually marked bumblebees for 20 to 41 days on a daily basis. All 48 bumblebees collected sugar solution at least on one day, and 41 individuals also collected pollen at least on one day. On average a worker collected sugar solution on 63% of the days, pollen on 6% of the days, both sugar solution and pollen on 15% of the days, and did not forage at all on 16% of the days (Fig. 4A). Adding all foraging tasks per worker for the entire period of time shows that only few bumblebees (15%) consistently collected only sugar solution, while the majority of bumblebees (85%) generally collected both sugar solution and pollen. None of the workers collected only pollen in the observation period (Fig. 4B). Counting the foraging flights of the 48 individual workers for 1 hour beginning at 11:00 on 5 different days showed that the workers made up to 11 foraging trips per hour (mean number of foraging trips/ hour  = 4.18±0.19 SE). No bumblebee collected only pollen during this one hour of observation.

#### Collective foraging on resources varying in quality

**Figure 5:**
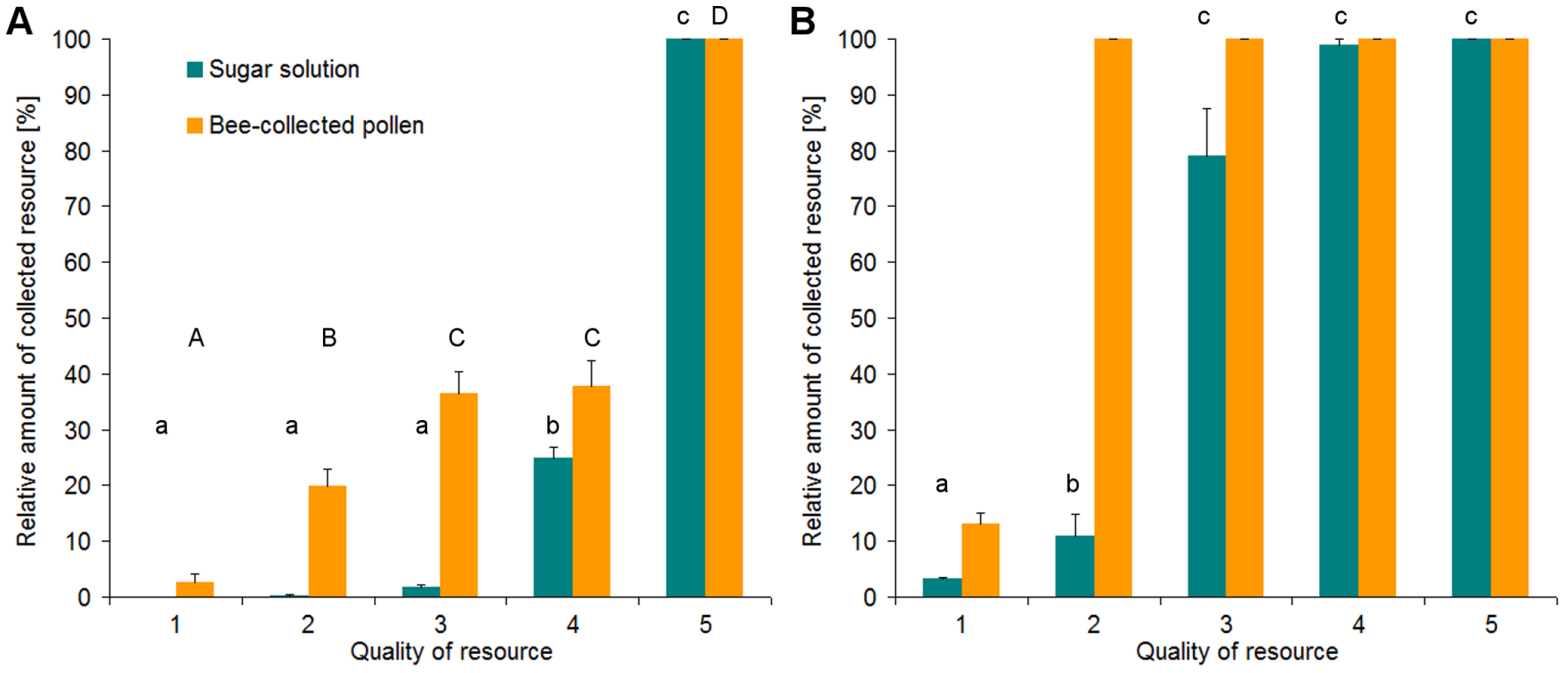
Average amount of resources collected from each feeder when the first feeder was emptied and when bees ceased foraging. **A**: Bumblebees emptied the first feeder: n = 76 trials with sugar solution; n = 12 trials with bee-collected pollen (Anova: F_4;355_ = 2.130,9; p<0,001 for sugar solution; F_4;55_ = 138,17; p<0,001 for pollen). **B**: Bumblebees stopped foraging: n = 11 trials with sugar solution; n = 3 trials with bee-collected pollen (Anova: F_4;105_ = 146,39; p<0,001 for sugar solution; no statistical analysis of data for pollen due to small sample size). Both sugar and pollen rewards were offered in 5 different qualities: sugar solution quality 1 = 0%; 2 = 9%; 3 = 18%; 4 = 27%; 5 = 36% sugar diluted in water; pollen quality 1 = 0%; 2 = 25%; 3 = 50%; 4 = 75%; 5 = 100% washed honeybee-collected pollen blended with dyed cellulose powder. Data presented are mean values and upper standard deviations. Different letters above columns indicate significant differences at p<0.001 from Tukey's Hsd-test.

This experiment showed that bumblebees significantly preferred the feeder offering the highest concentration of sugar solution as well as of pollen (Fig. 5A, Table 2). In all trials (n = 76), the first sugar solution feeder emptied contained the 36% solution. At this point in time, only 7% of the sugar solution from the other feeders had been collected. When bumblebees stopped foraging for sugar solution (n = 11 trials), they left 97% of the 0% sugar solution, 89% of the 9% sugar solution, 21% of the 18% sugar solution and 1% of the 27% sugar solution (Fig. 5B). In all trials (n = 12), the first pollen feeder emptied was offering 100% pollen. At this point in time, already 24% of the pollen/pollen surrogate from the other feeders had been collected. When bumblebees stopped foraging for pollen (n = 3), they only left 87% of the pure cellulose powder (i.e. 0% pollen: Fig. 5B).

**Table 2:** Statistical Analysis of the individual foraging on resource varying in quality and quantity experiment.

Figure	Series	rm Anova	P	Further test
6 A	Sugar solution	F_4;34_ = 35.1	<0.001	Paired t-tests
	Pollen	F_4;34_ = 0.4	0.818	-
6 B	Sugar solution	F_4;34_ = 11.8	0.001	Paired t-tests
	Pollen	F_4;34_ = 0.8	0.531	-
6 C	Sugar solution	F_4;38_ = 43.5	<0.001	Paired t-tests
	Pollen	F_4;38_ = 9.4	<0.001	Paired t-tests
6 D	Sugar solution	F_4;38_ = 37.9	<0.001	Paired t-tests
	Pollen	F_4;38_ = 2.6	0.051	-
7 A	Sugar solution	F_4;31_ = 2.2	0.093	-
	Pollen	F_4;31_ = 12.2	<0.001	Paired t-tests
7 B	Sugar solution	F_4;31_ = 0.6	0.681	-
	Pollen	F_4;31_ = 10.7	<0.001	Paired t-tests
7 C	Sugar solution	F_4;34_ = 0.3	0.911	-
	Pollen	F_4;34_ = 1.7	0.211	-
7 D	Sugar solution	F_4;34_ = 0.3	0.893	-
	Pollen	F_4;34_ = 2.5	0.051	-

**Figure 6:**
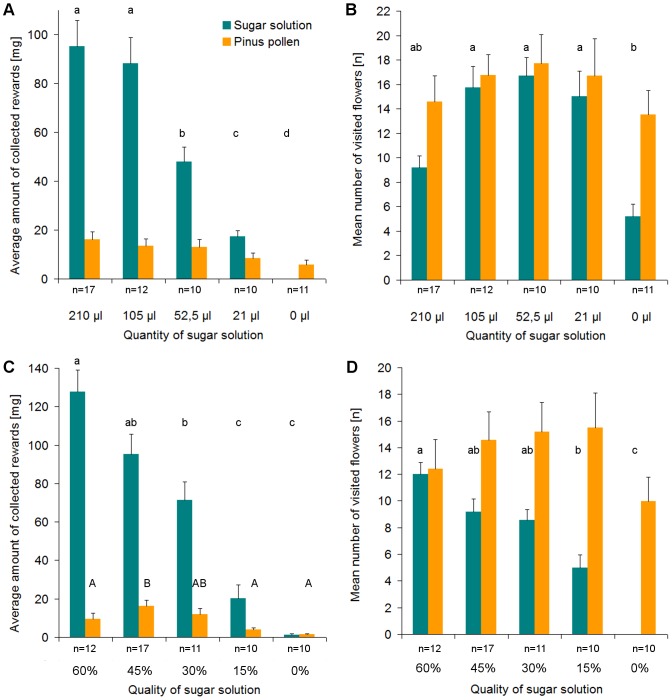
Nectar and pollen collection by individual bees when nectar rewards vary in quantity or quality. Collecting behaviour is defined by the amount of reward collected (**A**, **C**) and the number of flowers visited (**B**, **D**). In each trial, 21 flowers offered a specific amount and quality of pollen, and 21 flowers offered a specific amount and quality of sugar solution. Both types of rewards were evenly distributed among the 21 flowers, respectively. The pollen reward was always 1 ml of 100% *Pinus* pollen, offered simultaneously with either 1 of 5 different quantities of 45% sugar solution (**A**, **B**) or 1 of 5 different qualities of 210 μl sugar solution (**C**, **D**). n =  number of bumblebees tested per combination. Data presented are mean values and upper standard deviation. Different letters indicate significant differences at p<0.001 from pairwise comparisons using paired t-tests with Bonferroni correction.

**Figure 7:**
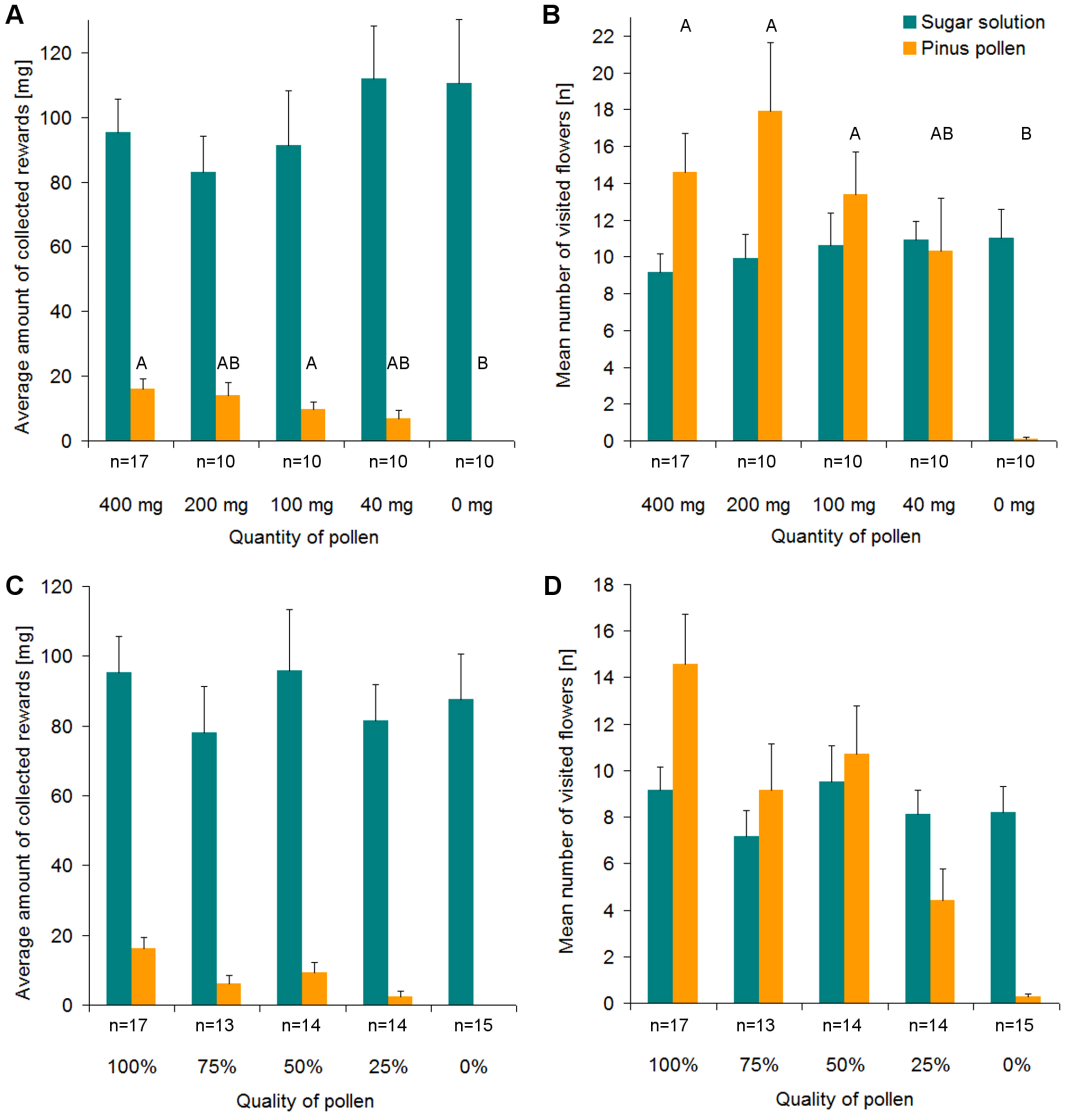
Nectar and pollen collection by individual bees when pollen rewards vary in quantity or quality. Collecting behaviour is defined by the amount of reward collected (**A**, **C**) and the number of flowers visited (**B**, **D**). In each trial, 21 flowers offered a specific amount and quality of pollen, and 21 flowers offered a specific amount and quality of sugar solution. Both types of rewards were evenly distributed among the 21 flowers, respectively. The nectar reward was always 210 μl of 45% sugar solution, offered simultaneously with either 1 of 5 different quantities of 100% *Pinus* pollen (**A**, **B**) or 1 of 5 different qualities of 1 ml *Pinus* pollen blended with glass powder (**C**, **D**). n =  number of bumblebees tested per combination. Data are mean values and upper standard deviation. Different letters indicate significant differences at p<0.001 from pairwise comparisons using paired t-tests with Bonferroni correction.

Results of the repeated measures Anova applied to the series of tests of individual foraging on resources varying in quality and quantity experiment shown in Figs. 6 and 7.

#### Individual foraging on resources varying in both quality and quantity

In this experiment the standard sugar solution reward (10 μl 45% sugar solution per flower) was collected by all of the 39 bumblebees tested in the series of tests with varying pollen reward. 22 out of 23 individuals collected the standard pollen reward (0.05 ml pure *Pinus* pollen per flower) in the series of tests with varying sugar solution reward. In the trial offering both the standard sugar solution and pollen reward (which is included in all four series of tests), 16 out of 17 bumblebees collected both sugar solution and pollen.

When the standard pollen reward was offered, both the amount of sugar solution collected and the number of sugar-rewarding flower visited depended on the quantity and quality of the reward offered (Fig. 6, Table 2). The amount of pollen collected was also affected by nectar and pollen rewards offered. Bumblebees exhibited a tendency to collect pollen instead of sugar solution, if the quantity of sugar solution reward was either extremely high or low. Particularly, in the absence of sugar solution reward, bumblebees collected less pollen. When the standard nectar reward was offered, the amount of pollen collected and the number of pollen-rewarding flowers visited correlated with the quantity and quality of the pollen available, but the nectar-foraging behaviour was not affected (Fig. 7).

## Discussion

The individual foraging tasks experiment indicates that only very few bumblebees specialize in collecting only one resource (sugar solution), with most workers gathering sugar solution and pollen. As the offer of food and storage levels were kept constant, the workers appeared to randomly collect nectar and/or pollen. Still there might be a certain pattern [47] – similar to the age polyethism tasks in honeybees [28] – if observed over a longer period. But unlike honeybees that store a large quantity of honey for the winter, bumblebees keep low storage levels to avoid theft of honey by mammals [27], which causes a need for flexibility in the foraging workers' tasks. Switching between different foraging tasks may be facilitated by using the same strategy for optimizing nectar and pollen foraging as recent studies with honeybees show that nectar- as well as pollen-foragers learn odour-mediated responses [48]. However, sucrose sensitivity may change during the time period in which honeybees collect nectar or pollen, causing an altered response to nectar rewards [14]. Recent studies on *Bombus impatiens* suggest that many foragers specialise to some degree in pollen or nectar foraging depending on the first foraging trips, but may switch if storage levels change [49]. A major methodological difference between our study and that of Hagbery and Nieh [49] is the pollen reward offered. Whereas in our study the pollen was washed with water and not ground, Hagbery and Nieh employed grinding of bee-collected pollen. The bumblebees might need less nectar if the regurgitated nectar from the honeybees' pollen collection is still covering the pollen. In addition the pollen might taste sweeter than pollen that has been washed and hence influence foraging decisions. Moreover substances that are shielded by the pollen wall in intact pollen grains might become accessible to bees in ground pollen.

The collective foraging on resources varying in quality experiment indicates that bumblebees are able to detect, and hence prefer, the highest quality of both sugar solution and pollen if different concentrations are offered simultaneously. However while bees effectively refused to collect sugar solution containing less than 10% sugar, they readily collected pollen from all feeders when blended with cellulose powder (they only left the pure cellulose powder in feeders). The results of Robertson et al. [50], who tested the bumblebees' ability to discriminate among plants on the basis of pollen quality (using *Mimulus guttatus* which is polymorphic for the percentage of cytoplasmless, inviable pollen grains), correspond with our findings. As long as pollen is abundant (due to low rates of visitation and filled feeders, respectively), the bees differentiate between pollen qualities, but when pollen becomes scarce (based on high density of foraging bees and decreasing amount of pollen in feeders, respectively), the bees cease to distinguish based on pollen quality. Mapalad et al. [44] have demonstrated that the thoracic temperature of bumblebee foragers is linked to pollen quality; however they used frozen pollen (probably collected by honeybees), which was ground without being washed suggesting that bees might have responded to the amount of regurgitated nectar present rather than the protein content. These authors also state that 25% and 50% pollen was rarely collected by bumblebees even without simultaneous offer of pollen of a higher quality [44]. This might be because they mixed bee-collected pollen and powdered cellulose by mass instead of volume – resulting in a smaller percentage of pollen per unit volume due to the much lower specific weight of cellulose powder compared to bee-collected pollen. As shown by Kitaoka and Nieh [25], pollen of different quality (mixture of washed bee-collected pollen and cellulose powder) positively influences the number of foragers exiting the nest. Their study also revealed that intranidal factors, like pollen storage levels and pollen odour, significantly affect pollen foraging. As the brood mainly determines the need for pollen, we made sure that pollen storage levels were consistently low to minimise the impact of these factors on bumblebee foraging decisions (see Material and Methods). Our results indicate that bumblebees follow different foraging rules for nectar and pollen collection. Whereas nectar foraging is focused on the highest concentrated source, pollen foraging includes all concentrations except the pure surrogate (cellulose powder with no pollen). The bumblebees' response to variation of pollen quality could be caused by either physical or chemical properties of the pollen / pollen-surrogate blend. Physical properties of the pollen blends were caused by the lower specific weight of cellulose powder compared to pollen and size and form of the cellulose particles; indeed we observed that the bumblebees needed more time and effort to collect pollen blends with a high cellulose percentage. Chemical properties of the pollen blends include olfactory and gustatory stimuli on the pollen surface as well as within the pollen grains. It is thus possible that bumblebees used the amount of olfactory and gustatory stimuli from the pollen surface as an indicator of pollen quality. We assume that bumblebees were unable to sense the protein content of pollen, which would require pollen consumption, breaking up pollen grains, and a protein taste receptor; however, we could not observe that the bumblebees feed on the pollen blends while collecting.

The pollen foraging behaviour seems to be shaped by the bumblebees' probable inability to sense the quality of pollen rewards as compared to the ability to sense the quality of nectar reward. It is unlikely that washing the honeybee-collected pollen prevented bumblebees from perceiving pollen quality as the protein content is locked inside the pollen grain [31]. Due to their limited ability to chemically sense the nutritional value of pollen [40], [51], bumblebees might rely on other sensory modalities such as vision. Indeed, Lunau [52], [53] regards the large number of plants that display visual pollen- and stamen-mimicking structures as related to the absence of chemical cues to identify pollen. In addition, if tactile cues were important for the recognition of pollen, foraging bumblebees might have been able to determine pollen quality, because pollen and cellulose powder are likely to feel different. However, since bumblebee workers regularly eat pollen [54], pollen foragers might be able to evaluate pollen quality. Observations of the gut content confirm pollen ingestion by bumblebees foraging for pollen in laboratory conditions (personal observations).

The individual foraging on resources varying in both quality and quantity experiment demonstrates that individual workers do not specialize in collecting either nectar or pollen as most bumblebees foraged for both resources (84.2%, 32 of 38, bees collected both rewards at least once when tested for varied amounts of rewards except for 0 μl sugar solution and 0 ml pollen; 15.8%, 6 of 38, bees collected only sugar solution). Bumblebee sugar solution and pollen collection behaviour probably has two components: first, the mean number of flowers visited shows bees' readiness to collect a certain reward and can be seen as a measure of attractiveness, and thus a measure of reward evaluation. Second, the bumblebees' evaluation of the reward is also indicated by the average amount of reward collected. Both factors are needed to infer the bumblebees' evaluation of the varying rewards as the number of visited flowers is no suitable measure when the quantity of a reward per flower is sufficiently high that the bee only needs to visit a small number of flowers to fill its corbiculae. Furthermore, the collected amount of rewards is not applicable if the quantity of reward offered is sufficiently small that the bee cannot collect enough to fill its corbiculae.

With decreasing quality of sugar solution, the average amount of pollen collected by the bumblebees is significantly smaller (Table 2), although the mean number of visits to flowers offering pollen did not alter significantly. This indicates that bees may need a certain minimum concentration of sugar solution to successfully package pollen into their corbiculae. Indeed, previous studies have shown that bumblebees regurgitate nectar for packaging pollen into their corbiculae [55]–[57].

The quantity of sugar solution or pollen does not alter the bumblebees' readiness to collect the reward, which can be explained by the fact that the quantities tested exceed the natural range of amount of reward per flower. As expected, the bees clearly distinguish between different qualities of sugar solution. Remarkably, workers tend to collect even more of the 60% sugar solution than of the 45% sugar solution. It should be expected that the bumblebees completely fill their honey sac when foraging for the 45% sugar solution as it already represents an increase as compared to the 30% sugar solution constantly offered in the feeding box. But it seems that – even while collecting a valuable reward – the bumblebees never cease looking out for even better reward. This strong preference might be related to individual searching behaviour [58] or communication among nestmates [59].

Our results show that foraging bumblebees respond more sensitively to variation in nectar quality than pollen quality. This suggests that bumblebees are either less able, or less motivated, to discriminate between different qualities of pollen compared to sugar solution. While it is doubtful whether bumblebees are able to appraise pollen quality because this is defined by its protein content and bees probably cannot assess this while foraging. This may be more relevant here since the artificial flowers used in this study were made to release the pollen by the bumblebees' buzzing. Moreover, pollen in natural flowers is not mixed with inert or nutrient-free substances and pseudopollen occurs only in very few species, such as *Maxillaria* orchids [60], whereas dilute nectar might be a more common phenomenon. Observed differences in the amounts of pollen blends collected could be due to the fact that the bees find it harder and/or less efficient to collect pollen when blends contain a higher ratio of cellulose powder:pollen. While the amount of 75% pollen collected is significantly smaller than the amount of 100% pollen collected, this does not clarify whether the difference is due to collecting efficiency or divergent evaluation of the rewards. But the number of visits, a measure for the bumblebees' readiness to collect a reward, does not differ significantly between 100% and 75% pollen, suggesting that bees do not discriminate between the two blends despite their disparity in quality. Considering the bees' apparent inability to sense pollen quality, how can a pollen-foraging bee be sure that the amount of pollen collected represents a sufficient provision for the development of larvae? Solitary bees may overprovision each nest cell so that even nutrient-poor pollen would provide enough protein for larval development. Alternatively, they may specialize in a (monophyletic) group of food plants, usually a family, in which the differences in pollen quality are less pronounced; this phenomenon has been described as oligolecty [61]. Alternatively, solitary bees may mix pollen from various food plants to provision each nest cell [62]. Furthermore, some social bees, including honeybees and bumblebees, do not provision each brood cell, but rather feed their larvae on demand [1] so that feeding of nutrient-poor pollen might be compensated by feeding a larger amount of pollen.

Although many factors such as initial experience [49], storage level [22] and quality of available food sources [25] seem to influence the foraging specialisations of individual bumblebees, it is largely unknown how bees assess the value of pollen sources. This study shows that individual bumblebees respond to changes in pollen quality, although this is less fine-tuned than the response to changes in nectar quality. The differences between the foraging rules for pollen and nectar may be highlighted as follows: nectar foraging focuses on nectar sources of the highest concentration, whereas pollen foraging includes all sources except pollen-free sources. Future studies will show whether pollen-foraging bees are unexpectedly able to sense pollen quality using visual, olfactory, gustatory or tactile cues, or even multimodal combinations of these cues, or whether they potentially use another indirect mechanism to assess pollen quality.
